# Serum uric acid to HDL-cholesterol ratio predicts all-cause mortality in cancer patients based on NHANES data

**DOI:** 10.1097/MD.0000000000048093

**Published:** 2026-03-20

**Authors:** Jiaying Zhang, Dong Niu, Valery Smirnov, Jiahui Li, Vladimir I. Gegechkori, Hengzhou Zhu, Xiaodan Zhu, Chunhui Jin

**Affiliations:** a Nanjing University of Chinese Medicine, Nanjing, Jiangsu, People’s Republic of China; b I.M. Sechenov First Moscow State Medical University (Sechenov University), Moscow, Russia; c Department of Oncology, Wuxi Affiliated Hospital of Nanjing University of Chinese Medicine, Wuxi, People’s Republic of China.

**Keywords:** all-cause mortality, cancer, HDL-cholesterol ratio, NHANES, serum uric acid

## Abstract

This study explores the relationship between serum uric acid to high-density lipoprotein-cholesterol ratio (UHR) and all-cause mortality among US cancer patients. Data from the 2005 to 2018 National Health and Nutrition Examination Survey, linked to National Death Index records until December 31, 2019, are analyzed. To investigate the relationship between UHR and mortality, multivariate Cox proportional hazards models are utilized. Kaplan–Meier curves compare survival across UHR quartiles, while the restricted cubic spline approach evaluates dose-response relationships. Subgroup analyses consider age, gender, diabetes, hypertension, body mass index, and income-to-poverty ratio, with stratified analyses by cancer type. Two sensitivity analyses exclude participants under 65 and those with multiple cancers. During the 81.0-month follow-up period, the all-cause mortality rate is 15.6%. Cancer patients in the top quartile group have a significantly increased mortality risk (hazard ratio: 1.43, 95% confidence interval: 1.07–1.93). Kaplan–Meier survival analysis links higher UHR to reduced survival. This association is nonlinear and more pronounced in those aged ≥ 65, males, and individuals with diabetes or hypertension. Stratified analyses reveal a significant link in breast, colorectal, and prostate cancers. UHR may serve as a valuable predictor of all-cause mortality among individuals with cancer.

## 1. Introduction

Cancer is one of the leading causes of death worldwide.^[1]^ Since 1990, the mortality rate among cancer patients has increased sharply, climbing from the 3rd to the 2nd most common cause of death by 2013, surpassed only by heart disease.^[2]^ By the end of 2025, the United States is expected to report 2,041,910 new cases of cancer and 618,120 deaths from this disease.^[3]^ Cancer is still a leading cause of high mortality rates, despite tremendous progress in its prevention, diagnosis, and treatment.^[4]^ Cancer sufferers face not only the physical pain and suffering brought on by the illness and its treatment, but also the financial burden of expensive medical costs. Identifying effective prognostic biomarkers early in the disease could help identify high-risk patients and enable targeted intervention strategies. This approach would not only alleviate healthcare costs and reduce patient suffering, but also improve treatment outcomes and significantly enhance overall survival (OS) rates among cancer patients.^[5]^ Therefore, exploring potential prognostic biomarkers for cancer and conducting early intervention studies hold profound clinical significance and societal value.

Higher mortality rates among cancer patients and the incidence of different types of cancer are positively connected with elevated serum uric acid (UA) levels. By encouraging processes like oxidative stress, elevated UA levels may raise the risk of cancer-related mortality.^[6]^ Evidence indicates that elevated UA concentrations are connected with lower survival rates in patients with breast cancer^[7]^ and that they are linked to a higher incidence of kidney cancer in women.^[8]^ Meanwhile, increased high-density lipoprotein-cholesterol (HDL-C) is inversely associated with disease risk. HDL-C is widely recognized as a potential biomarker for disease prevention.^[9]^ According to studies, HDL-C is sufficient to reduce the formation of an inflammatory microenvironment, which may help reduce the risk of cancer development or death.^[10]^

However, UA and HDL-C levels are influenced by renal excretion and lipid metabolism.^[11–13]^ Compared to individual UA or HDL-C indicators, the serum uric acid to high-density lipoprotein-cholesterol ratio (UHR) has been increasingly incorporated into joint research in recent years. By integrating the pro-oxidant properties of elevated UA with the diminished antiinflammatory capacity of low HDL-C, UHR mitigates the variability of isolated indicators and provides a highly stable reflection of systemic metabolic and inflammatory status.^[14]^ Accordingly, elevated UHR is an established predictor for cardiovascular diseases (CVD), such as atherosclerosis^[15]^ and acute coronary syndrome,^[16]^ primarily due to its strong correlation with endothelial dysfunction and subclinical inflammation.^[17,18]^ Despite this extensive validation in cardiovascular research, the prognostic utility of UHR in cancer remains notably underexplored. Although traditionally classified as distinct clinical entities, CVD, and cancer exhibit significant pathophysiological overlap. Beyond shared clinical risk factors, including hypertension, hyperlipidemia, obesity, and diabetes,^[19–22]^ both conditions are fundamentally driven by common mechanisms such as chronic low-grade inflammation, oxidative stress, and metabolic dysregulation.^[23]^ In this context, an elevated UHR signifies a deleterious systemic microenvironment characterized by excessive reactive oxygen species and inflammasome activation. These pathological states not only accelerate vascular damage but also promote tumor cell proliferation, genomic instability, and immune evasion.^[24]^

As a combination of multiple physiologic processes, including lipid metabolism, inflammatory response, and endothelial function, the potential impact of UHR on the mortality of cancer patients warrants further exploration. To determine the degree of the relationship between UHR and all-cause death risk in cancer patients, this study used data from the National Health and Nutrition Examination Survey (NHANES).

## 2. Approaches

### 2.1. Population and research design

The National Center for Health Statistics performed the NHANES survey, which provided the data for this cross-sectional research. Through questionnaires and medical exams, the survey gathers information on demographics, nutrition, physiology, and laboratory results. A nationally representative sample design was employed to ensure sample diversity, incorporating complex stratified, multi-stage, probability sampling, with biennial surveys conducted. The National Center for Health Statistics Research Ethics Review Committee approved the study and signed informed consent papers to guarantee that participants rights were protected. The website (https://wwwn.cdc.gov/nchs/nhanes/) provides access to the NHANES datasets, including the one utilized in this study.

**Figure 1.**
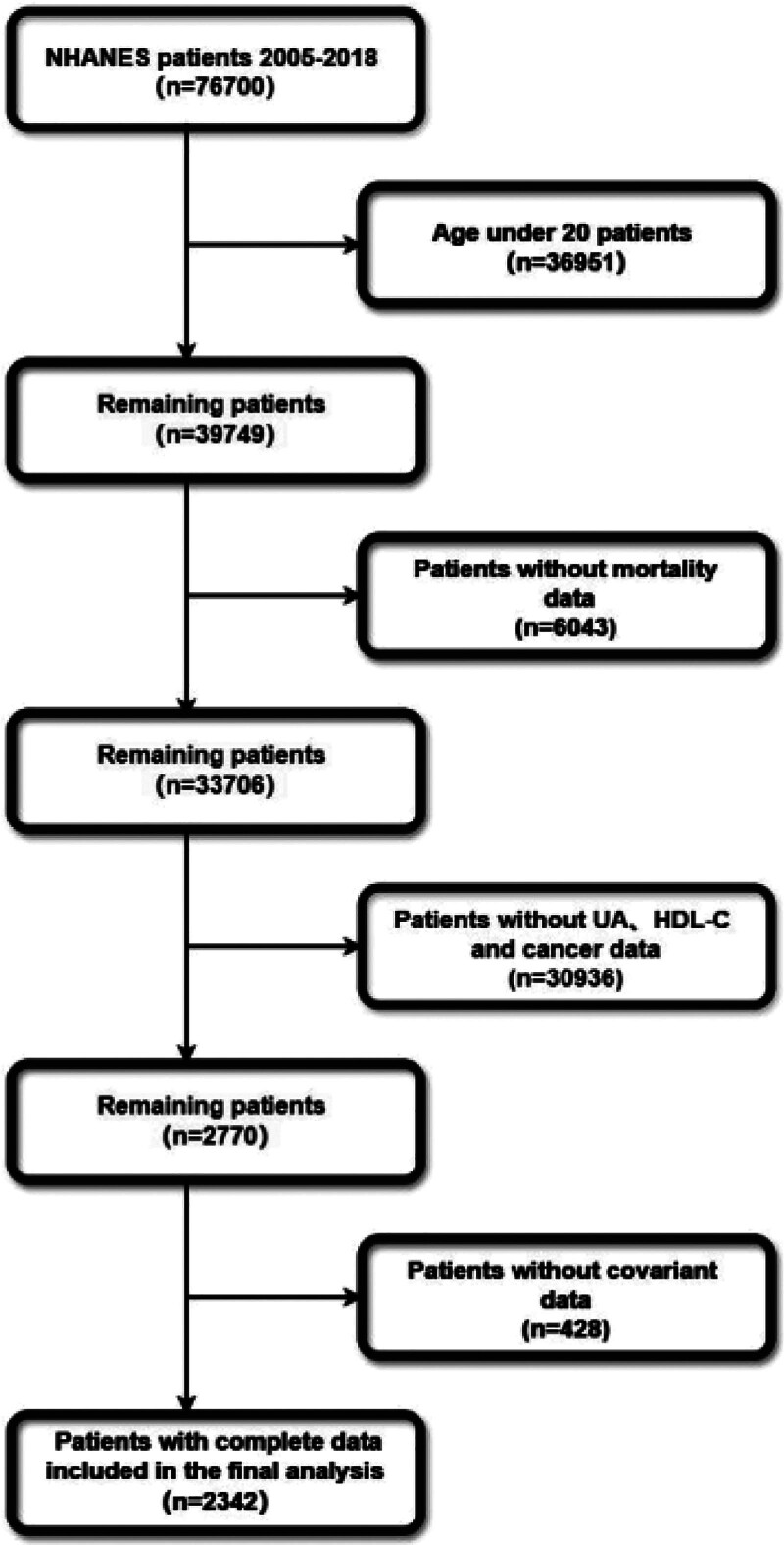
The participants’ selection flow chart.

Cancer data were obtained through self-reported or proxy-reported interviews. To ensure diagnostic accuracy, participants were identified as having cancer if they answered “yes” to the question: “Has a doctor or other health professional ever told you that you had cancer or a malignancy of any kind?.” All included participants had been informed by a physician or healthcare provider that they had cancer or any other form of malignancy, including the type and number of cancers. This study focused on 76,700 participants from the NHANES 2005 to 2018 study cycles. The following exclusion criteria were applied: age under 20; no information on mortality status; incomplete HDL-C or UA data, or no cancer diagnosis; incomplete covariate data. Finally, 2342 cancer patients were part of this research (Fig. 1). It is worth noting that participants under 20 were excluded from the analysis for 2 primary reasons. First, this aims to minimize the confounding effects of adolescent-related hormonal changes on metabolic indicators, as significant differences exist between adolescents and adults in these metrics. Second, the focus is on malignancies occurring in adulthood, as childhood cancers exhibit distinct biological and etiological characteristics.

### 2.2. The UHR computation

The ratio of serum UA (mg/dL) to HDL-C (mg/dL) was used to compute the exposure variable UHR. A multi-channel analyzer used the timed endpoint method to evaluate serum UA levels, while a photometric technique was used to assess HDL-C levels directly. Specific laboratory procedures can be found on the NHANES website. Given the absence of a universally accepted clinical cutoff for UHR in cancer prognosis, this study employed quartiles to objectively explore potential nonlinear associations. Compared to tertiles or quintiles, quartiles enabled finer risk gradient identification while ensuring sufficient mortality events across subgroups to maintain statistical robustness. Participants were categorized into 4 groups (Q1–Q4) based on the weighted quartiles of the UHR distribution.

### 2.3. Outcome determination

The NHANES Public Use Linked Mortality file, which was updated as of December 31, 2019, was used to calculate the mortality status. Probabilistic matching algorithms were used to link it to National Death Index data.^[25]^ The difference between the date of the baseline examination and the most recent date of known survival or death was referred to as the follow-up time. See the website (https://www.cdc.gov/nchs/linked-data/mortality-files/index.html) for more specific information about mortality data.

### 2.4. Covariates

This investigation took into account the following potential confounders. Among the demographic factors are age, gender, race, marital status, education level, and income-to-poverty ratio (PIR). Race was divided into Mexican American, nonHispanic White, nonHispanic Black, and other races. Less than high school, high school, college, or above were the categories for education. There were 3 categories for marital status: married, divorced, and unmarried. Total cholesterol and body mass index (BMI) were among the clinical measurements. Questionnaire data included general health status, health insurance, hypertension, coronary heart disease, and diabetes.^[17]^ Please visit the NHANES website for comprehensive information on covariates.

### 2.5. Analysis of statistics

Considering the complex sampling design of NHANES, statistical analyses were conducted using appropriate weights and stratification variables (*E*). Baseline characteristics were described according to the UHR quartiles (Q1–Q4) of participants. The use of quartiles ensures that the study possesses adequate statistical power to detect significant associations in the highest-risk stratum without compromising the precision of the hazard ratio (HR) estimations. Continuous variables were represented as mean ± standard deviation. Group differences were examined using the Mann–Whitney *U* test for nonnormally distributed data and 1-way analysis of variance for regularly distributed data. The chi-square test was used to compare group differences, and categorical variables were represented as percentages.

Using 4 models to account for confounders, a multivariate weighted Cox proportional hazards regression model was used to evaluate the independent predictive value of UHR. Potential confounding factors were selected based on biological plausibility and prior literature, focusing on key variables associated with both the exposure factors and the outcome measure. The fully adjusted model included demographic characteristics (age, sex, ethnicity), socioeconomic indicators (health insurance status as a proxy for healthcare accessibility), and clinical characteristics (cancer type). Model 1 was left unaltered, Model 2 was age and gender adjusted, Model 3 had age, gender, race, and health insurance adjusted, and Model 4 had age, gender, race, health insurance, and cancer type adjusted. Mortality risk across UHR quartile groups was assessed using Kaplan–Meier survival curves. The dose-response connection between UHR levels and all-cause mortality in cancer patients was shown using restricted cubic spline (RCS). To improve the reliability and stability of this study, subgroup analyses were performed according to age (< 65 years, ≥ 65 years), gender (male, female), diabetes (yes, no, borderline), hypertension (yes, no), BMI (≤ 25, 25–30, ≥ 30 kg/m^2^), and PIR (≤ 1.71, 1.71–3.76, and ≥ 3.76). To investigate whether specific cancer patient groups were more likely to benefit from changes in UHR, we stratified the association between UHR and all-cause mortality across various cancer types using a weighted Cox proportional hazards regression model. The subgroup and stratified analysis sections were also subjected to false discovery rate (FDR) correction to account for the possibility of false positives in multiple hypothesis testing. All *P* values were derived from the Cox regression model, and the Benjamini–Hochberg technique was used to adjust for FDR.^[26]^ Finally, a sensitivity analysis was conducted by removing participants under 65 to rule out the potential that the long survival effect in younger patients would diminish the predictive impact of UHR as a risk factor. Additionally, to control for the confounding effect of the more complex physiological conditions in patients with multiple cancers, a 2nd sensitivity analysis was also carried out by eliminating participants who had multiple cancers (2 or more). Statistical significance was defined as a *P* value <.05. R (version 4.4.1; R Foundation for Statistical Computing, Vienna, Austria) and SPSS (version 27.0; IBM Corp., Armonk) were used for all analyses.

## 3. Results

### 3.1. Baseline features of participants

The UHR quartiles were used to group the 2342 subjects. Participants were 42.7% male and 57.3% female, with an average age of 62.78 ± 0.40 years. The mean UHR (± standard deviation) was 11.12 ± 0.13. Compared to the other quartiles, participants in the Q4 group were more likely to be male, older, and to have a higher BMI, showing a higher risk of mortality. Participants in the Q4 group showed greater prevalence of bladder, colorectal, and lung cancers than those in the Q1 group, and breast cancer had the highest percentage of all cancer types. With the exception of race, marital status, and health insurance, all other variables exhibited significant differences in distribution (*P *< .05). Details are in Table 1.

**Table 1:** Participants’ baseline features.

	Overall	UHR quartile groups				*P*
		≤7.65	7.65–10.58	10.58–14.39	>14.39	
N	20,190,240.3	5,720,945.1	4,643,592.4	5,157,494.3	4,668,208.5	
Gender (%)						<.001
Male	8,627,464.2 (42.7)	842,049.3 (14.7)	1,654,283.3 (35.6)	2,733,774.4 (53.0)	3,397,357.3 (72.8)	
Female	11,562,776.1 (57.3)	4,878,895.8 (85.3)	2,989,309.2 (64.4)	2,423,719.9 (47.0)	1,270,851.1 (27.2)	
Age (yr)	62.78 ± 0.40	61.21 ± 0.73	62.99 ± 0.65	63.60 ± 0.70	63.60 ± 0.72	<.001
Race (%)						.204
Mexican-American	485,947.6 (2.4)	150,279.5 (2.6)	120,659.5 (2.6)	100,300.6 (1.9)	114,707.9 (2.5)	
Nonhispanic White	17,542,102.5 (86.9)	5,039,603.5 (88.1)	3,995,367.7 (86.1)	4,582,218.9 (88.9)	3,924,912.4 (84.1)	
Nonhispanic Black	933,569.3 (4.6)	209,442.2 (3.7)	248,112.1 (5.3)	231,809.6 (4.5)	244,205.4 (5.2)	
Other races	1,228,620.9 (6.1)	321,619.8 (5.6)	279,453.1 (6.0)	243,165.2 (4.7)	384,382.8 (8.2)	
Education (%)						.028
Less than high school	2,130,054.2 (10.6)	523,017.0 (9.1)	492,249.9 (10.6)	421,856.6 (8.2)	692,930.6 (14.8)	
High school	4,143,989.2 (20.5)	1,031,921.1 (18.1)	995,673.7 (21.4)	1,114,376.3 (21.6)	1,002,018.1 (21.5)	
College or above	13,916,196.9 (68.9)	4,166,007.0 (72.8)	3,155,668.7 (68.0)	3,621,261.3 (70.2)	2,973,259.8 (63.7)	
Marriage (%)						.275
Married	15,749,624.7 (78.0)	4,377,957.8 (76.5)	3,569,912.6 (76.9)	4,014,877.7 (77.8)	3,786,876.5 (81.1)	
Divorce	2,725,214.9 (13.5)	859,194.3 (15.0)	591,463.0 (12.7)	788,143.7 (15.3)	486,413.9 (10.4)	
Unmarried	1,715,400.7 (8.5)	483,793.0 (8.5)	482,216.8 (10.4)	354,472.9 (6.9)	394,918.0 (8.5)	
PIR	3.35 ± 0.05	3.53 ± 0.08	3.20 ± 0.08	3.38 ± 0.09	3.27 ± 0.09	<.001
UHR (%)	11.12 ± 0.13	5.77 ± 0.08	9.15 ± 0.04	12.26 ± 0.06	18.37 ± 0.20	<.001
UA (mg/dL)	5.47 ± 0.04	4.16 ± 0.06	5.20 ± 0.05	5.86 ± 0.04	6.92 ± 0.07	<.001
HDL (mg/dL)	55.24 ± 0.56	73.93 ± 0.88	57.22 ± 0.58	47.96 ± 0.36	38.42 ± 0.43	<.001
TC (mg/dL)	195.58 ± 1.65	207.45 ± 2.03	198.74 ± 2.36	190.34 ± 3.47	183.70 ± 4.00	<.001
BMI (kg/m^2^)	29.20 ± 0.16	25.68 ± 0.31	28.94 ± 0.30	30.66 ± 0.36	32.16 ± 0.35	<.001
Cancer type*						<.001
Bladder	374,885.5 (1.9)	76,666.8 (1.3)	69,429.8 (1.5)	113,964.2 (2.2)	114,824.8 (2.5)	
Blood	54,064.6 (0.3)	0	20,569.9 (0.4)	8763.1 (0.2)	24,731.6 (0.5)	
Bone	47,059.7 (0.2)	1464.6 (0.0)	1145.1 (0.0)	39,248.2 (0.8)	5201.8 (0.1)	
Brain	64,804.5 (0.3)	12,388.2 (0.2)	13,449.3 (0.3)	22,597.5 (0.4)	16,369.4 (0.4)	
Breast	3,199,992.8 (15.8)	1,272,337.9 (22.2)	777,792.5 (16.7)	820,316.9 (15.9)	329,545.6 (7.1)	
Cervix (cervical)	1,555,751.1 (7.7)	544,082.0 (9.5)	528,201.9 (11.4)	329,262.1 (6.4)	154,205.2 (3.3)	
Colorectum	950,663.3 (4.8)	256,092.6 (4.5)	203,251.5 (4.4)	232,452.4 (4.5)	258,866.8 (5.5)	
Esophagus	66,215.3 (0.3)	14,550.1 (0.3)	7883.8 (0.2)	18,661.9 (0.4)	25,119.6 (0.5)	
Kidney	311,120.2 (1.5)	40,453.6 (0.7)	79,843.9 (1.7)	67,890.3 (1.3)	122,932.4 (2.6)	
Leukemia	197,423.9 (1.0)	23,069.1 (0.4)	34,197.5 (0.7)	81,335.2 (1.6)	58,822.0 (1.3)	
Liver	69,610.2 (0.3)	6020.9 (0.1)	27,448.2 (0.6)	29,241.2 (0.6)	6899.9 (0.1)	
Lung	261,967.3 (1.3)	45,146.3 (0.8)	65,926.2 (1.4)	46,794.7 (0.9)	104,100.1 (2.2)	
Lymphoma/Hodgkin disease	366,466.1 (1.8)	86,234.6 (1.5)	117,337.6 (2.5)	85,520.1 (1.7)	386,695.4 (1.7)	
Melanoma	1,556,642.9 (7.7)	460,779.6 (8.1)	243,423.6 (5.2)	465,744.3 (9.0)	386,695.4 (8.3)	
Mouth/tongue/lip	127,699.3 (0.6)	61,842.8 (1.1)	33,265.5 (0.7)	0	32,591.1 (0.7)	
Ovary	306,999.2 (1.5)	70,415.3 (1.2)	101,111.9 (2.2)	58,971.1 (1.1)	76,500.9 (1.6)	
Pancreas	13,815.1 (0.1)	3138.0 (0.1)	6568.8 (0.1)	4108.3 (0.1)	0	
Prostate	1,900,492.3 (9.4)	164,444.8 (2.9)	455,266.0 (9.8)	587,127.8 (11.4)	693,653.6 (14.9)	
Skin (nonmelanoma)	4,432,480.9 (22.0)	1,385,793.1 (24.2)	862,827.5 (18.6)	1,131,472.9 (21.9)	1,052,387.4 (22.5)	
Skin (don’t know what kind)	1,777,032.0 (8.8)	394,537.0 (6.9)	425,491.9 (9.2)	501,815.8 (9.7)	455,187.4 (9.8)	
Soft tissue (muscle or fat)	32,576.1 (0.2)	15,246.6 (0.3)	0	0	17,329.4 (0.4)	
Stomach	74,180.7 (0.4)	53,339.0 (0.9)	5678.6 (0.1)	10,383.9 (0.2)	4779.1 (0.1)	
Testis	253,373.0(1.3)	11,309.2 (0.2)	18,941.7 (0.4)	64,290.4 (1.2)	158,831.7 (3.4)	
Thyroid	440,544.9 (2.2)	180,071.9 (3.1)	116,392.3 (2.5)	80,520.5 (1.6)	63,560.1 (1.4)	
Uterus	662,438.4 (3.3)	238,243.4 (4.2)	179,400.5 (3.9)	158,507.7 (3.1)	86,286.8 (1.8)	
Other	1,022,174.0 (5.1)	303,277.8 (5.3)	242,854.3 (5.2)	189,975.4 (3.7)	286,066.4 (6.1)	
2 types of cancer (%)	2,300,336.7 (11.4)	661,364.0 (11.6)	531,195.6 (11.4)	627,700.7 (12.2)	480,076.4 (10.3)	.048
3 types of cancer (%)	279,634.6 (1.4)	63,085.0 (1.1)	84,864.2 (1.8)	105,082.4 (2.0)	26,603.0 (0.5)	.008
Hypertension (%)						<.001
Yes	10,436,210.5 (51.7)	2,249,876.5 (39.3)	2,098,544.8 (45.2)	2,828,431.1 (54.8)	3,259,358.1 (69.8)	
No	9,754,029.8 (48.3)	3,471,068.6 (60.7)	2,545,047.7 (54.8)	2,329,063.2 (45.2)	1,408,850.4 (30.2)	
Coronary heart disease (%)						<.001
Yes	1,617,263.8 (8.0)	252,680.1 (4.4)	321,410.1 (6.9)	361,998.4 (7.0)	681,175.2 (14.6)	
No	18,572,976.5 (92.0)	5,468,265.0 (95.6)	4,322,182.4 (93.1)	4,795,496.0 (93.0)	3,987,033.3 (85.4)	
Diabetes (%)						<.001
Yes	3,361,848.7 (16.6)	397,912.1 (7.0)	615,672.7 (13.2)	1,009,438.0 (19.6)	1,338,825.7 (28.7)	
Borderline	724,219.8 (3.6)	157,272.0 (2.7)	207,378.5 (4.5)	179,893.3 (3.5)	179,676.1 (3.8)	
No	16,104,171.8 (79.8)	5,165,761.0 (90.3)	3,820,541.2 (82.3)	3,968,163.0 (76.9)	3,149,706.6 (67.5)	
Health insurance (%)						.129
Yes	19,097,222.9 (94.6)	5,318,658.8 (93.0)	4,337,664.9 (93.4)	4,946,754.3 (95.9)	4,494,144.8 (96.3)	
No	1,093,017.4 (5.4)	402,286.3 (7.0)	305,927.5 (6.6)	210,740.0 (4.1)	174,063.7 (3.7)	
General health condition (%)						<.001
Good	16,105,564.5 (79.8)	4,999,378.6 (87.4)	3,751,724.8 (80.8)	4,096,417.4 (79.4)	3,258,043.6 (69.8)	
Fair	3,375,459.3 (16.7)	622,438.2 (10.9)	725,305.6 (15.6)	850,857.3 (16.5)	1,176,858.2 (25.2)	
Poor	709,216.5 (3.5)	99,128.4 (1.7)	166,562.0 (3.6)	210,219.6 (4.1)	233,306.6 (5.0)	

BMI = body mass index, PIR = income-to-poverty ratio, TC = total cholesterol, UA = serum uric acid, UHR = serum uric acid to HDL-cholesterol ratio.

* Minor percentages of participants selected “Refused” or “Don’t know,” therefore the total did not add up to 100%.

### 3.2. Associations between UHR and mortality from all causes

During the follow-up period, a total of 552 deaths were observed, representing a weighted total of 3,159,010 deaths in the population. The median follow-up time for the entire cohort was 81.0 months (95% confidence interval [CI]: 79.0–85.0). Notably, the mortality rate showed a significant upward trend, increasing from 12.5% in the Q1 group to 21.6% in the Q4 group. We further explored the relationship between UHR and its interquartile groupings with all-cause death in cancer patients by constructing 4 multivariate weighted Cox proportional hazards regression models (Table 2).

**Table 2:** Relationships between UHR and all-cause mortality*.

No. of deaths (weighted %)†		Model 1			Model 2			Model 3			Model 4		
		HR	95% CI	*P*	HR	95% CI	*P*	HR	95% CI	*P*	HR	95% CI	*P*
UHR	552 (15.6%)	1.23	1.11–1.36	<.001	1.13	1.01–1.26	.0206	1.13	1.02–1.26	.0187	1.03	1.01–1.05	<.001
Q1	110 (12.5%)	Reference											
Q2	127 (15.3%)	1.09	0.80–1.49	.5970	0.90	0.67–1.21	.4942	0.90	0.68–1.21	.4935	0.92	0.68–1.25	.610
Q3	132 (14.1%)	1.15	0.84–1.56	.3890	0.90	0.66–1.21	.4770	0.90	0.66–1.22	.5021	0.93	0.68–1.28	.670
Q4	183 (21.6%)	1.88	1.39–2.55	<.001	1.43	1.06–1.93	.0180	1.43	1.07–1.93	.0168	1.45	1.06–2.00	.021
*P* for trend				<.001			<.001			<.001			<.001

Model 1 was left unaltered.

Model 2 had age and gender adjusted.

Model 3 had age, gender, race, and health insurance adjusted.

Model 4 had age, gender, race, health insurance, and cancer type adjusted.

CI = confidence interval, HR = hazard ration, UHR = serum uric acid to HDL-cholesterol ratio.

* Four multivariate Cox proportional hazards regression models to control for confounding factors.

† “No. of deaths” represents the unweighted number of events in the study sample, while percentages (%) are weighted estimates representing the US population.

The unadjusted model (Model 1) showed a significant correlation between high UHR levels and cancer patients all-cause death (HR = 1.23, 95% CI: 1.11–1.36, *P* < .001). When grouped by UHR quartiles, the highest quartile (Q4) showed a significantly higher risk of death than the reference group (Q1) (HR = 1.88, 95% CI: 1.39–2.55, *P* < .001). UHR levels and all-cause mortality in cancer patients were found to be significantly correlated linearly (*P* for trend < .001) by trend analysis.

UHR was still substantially linked to all-cause mortality in cancer patients in the model that adjusted for age and gender (Model 2) (HR = 1.13, 95% CI: 1.01–1.26, *P* = .0206). The Q4 group had a significant linear trend (*P* for trend = .0180) and an elevated risk of death (HR = 1.43, 95% CI: 1.06–1.93, *P* = .018).

In a model that further adjusted for age, gender, race, and health insurance (Model 3), the association remained significant (HR = 1.13, 95% CI: 1.02–1.26, *P* = .0187). Similarly, the Q4 group showed a significant risk of death (HR = 1.43, 95% CI: 1.07–1.93, *P *= .0168), and the test for trend was significant (*P* for trend = .0168).

To determine whether the observed association was driven by specific high-mortality cancer types, we further adjusted cancer type based on Model 3 (Model 4). The results showed that UHR remained a robust and independent predictor of mortality. The Q4 group maintained a significantly elevated risk of death (HR = 1.45, 95% CI: 1.06–2.00, *P* = .021) compared to the Q1 group, confirming that the prognostic value of UHR is independent of the specific malignancy type.

**Figure 2.**
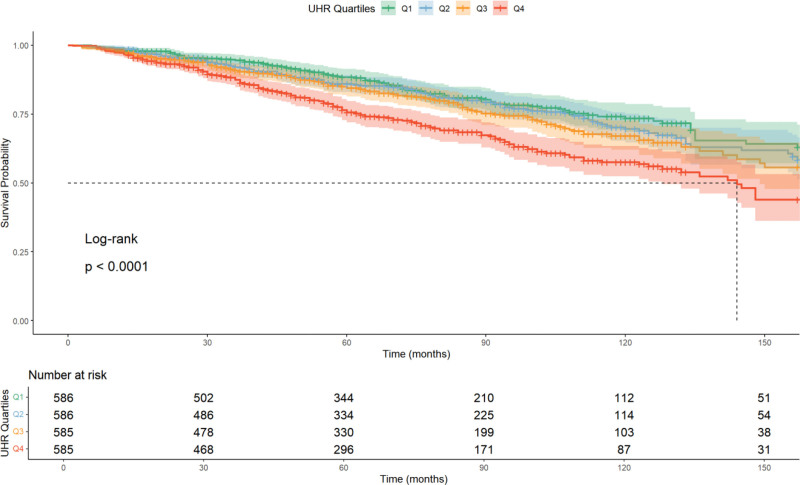
The Kaplan–Meier survival curve and risk table for cancer patients’ all-cause death. UHR = serum uric acid to HDL-cholesterol ratio.

Kaplan–Meier survival analysis was conducted, as shown in Figure 2. We compared the survival distributions among the 4 UHR groups using the Log-rank test. The results demonstrated a statistically significant difference in OS across the quartiles (Log-rank *P* < .001). Notably, the median OS was 144.0 months (95% CI: 128.0–165.0) in the Q4 group, whereas it was not reached in the Q1, Q2, and Q3 groups. This indicates that patients with the highest UHR levels have a significantly accelerated mortality risk compared to those with lower levels, reinforcing UHR as a potential biomarker for poor prognosis in cancer patients.

### 3.3. Nonlinear association of UHR with all-cause mortality

**Figure 3.**
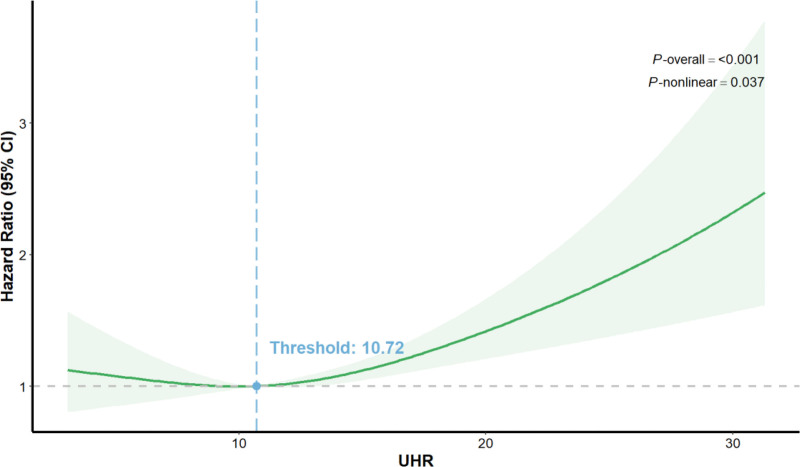
Restricted cubic spline of UHR and all-cause mortality in cancer patients (HR: line; 95% CI: green shading). CI = confidence interval, HR = hazard ratio, UHR = serum uric acid to HDL-cholesterol ratio.

Cox regression analyses indicated a nonlinear relationship between UHR and all-cause mortality in cancer patients. A RCS model was used to further show the dose-response association of UHR with all-cause mortality in cancer patients (Fig. 3). A nonlinear connection between UHR and cancer patients all-cause mortality was observed after controlling for potential confounders(*P* nonlinear = .037). The mortality risk remained relatively stable at lower UHR levels. However, a distinct threshold was identified at a UHR of 10.72, beyond which the HR rose steeply above 1.0, indicating a rapidly escalating risk of death with further UHR increases.

### 3.4. Results of subgroup analyses

**Figure 4.**
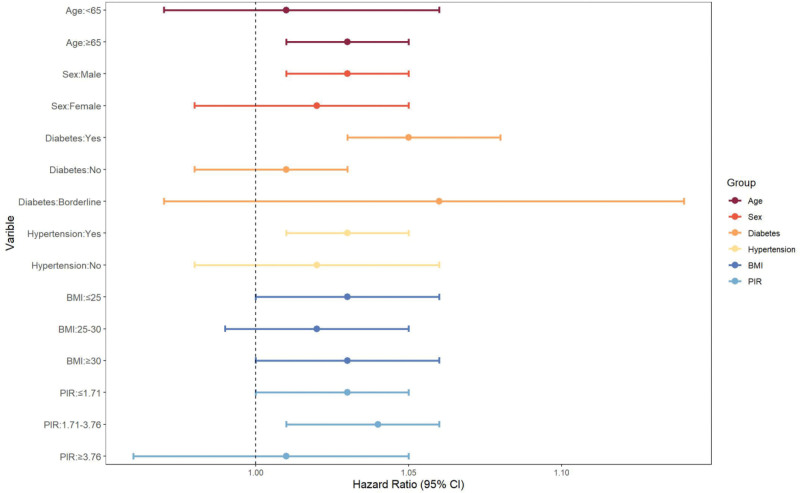
Forest plot of subgroup analyses between UHR and all-cause mortality. CI = confidence interval, UHR = serum uric acid to HDL-cholesterol ratio.

We performed subgroup analyses based on age (<65 years, ≥65 years), gender (male, female), diabetes (yes, no, borderline), hypertension (yes, no), BMI (≤ 25, 25–30, ≥30 kg/m^2^), and PIR (≤ 1.71, 1.71–3.76, ≥ 3.76) to assess the stability of the association between UHR and all-cause mortality of cancer patients. FDR approach was used to modify the *P* values (Table 3, Fig. 4). Results showed that risk increases were concentrated in specific subgroups (≥65 years, men, diabetics, and hypertensive patients). Specifically, in the analysis stratified by age, individuals ≥ 65 years of age had a significantly increased risk compared with those < 65 years of age (HR = 1.03, 95% CI: 1.01–1.05, *P*_adjusted < .001). The mortality risk was significantly higher in males than in females (HR = 1.03, 95% CI: 1.01–1.05, *P*_adjusted = .017). Diabetic patients had a significantly increased risk (HR = 1.05, 95% CI: 1.03–1.08, *P*_adjusted = .001), whereas the risk for those without diabetes or in a diabetic borderline status did not change significantly. Individuals with hypertension were at a considerably higher risk (HR = 1.03, 95% CI: 1.01–1.05, *P*_adjusted = .008), while those without hypertension did not show statistical significance. In the BMI and PIR subgroups, none of the FDR-adjusted *P* values reached the significance threshold (*P*_adjusted < .05).

**Table 3:** Subgroup analyses of UHR and all-cause mortality.

Variable	Count	Percent	HR (95% CI)	*Praw* value	*P_adjusted* value (FDR)
Age					
<65	985	42.1	1.01 (0.97–1.06)	.589	.631
≥65	1357	57.9	1.03 (1.01–1.05)	<.001	.007
Sex					
Male	1106	47.2	1.03 (1.01–1.05)	.005	.017
Female	1236	52.8	1.02 (0.98–1.05)	.328	.419
Diabetes					
Yes	464	19.8	1.05 (1.03–1.08)	<.001	.001
No	1785	76.2	1.01 (0.98–1.03)	.582	.631
Borderline	93	4.0	1.06 (0.97–1.14)	.190	.286
Hypertension					
Yes	1358	58.0	1.03 (1.01–1.05)	.002	.008
No	984	42.0	1.02 (0.98–1.06)	.335	.419
BMI					
≤25	650	27.8	1.03 (1.00–1.06)	.073	.137
25–30	806	34.4	1.02 (0.99–1.05)	.177	.286
≥30	886	37.8	1.03 (1.00–1.06)	.035	.076
PIR					
≤1.71	785	33.5	1.03 (1.00–1.05)	.035	.076
1.71–3.76	776	33.1	1.04 (1.01–1.06)	.006	.017
≥3.76	781	33.3	1.01 (0.96–1.05)	.761	.761

CI = confidence interval, FDR = false discovery rate, HR = hazard ration, PIR = income-to-poverty ratio, UHR = serum uric acid to HDL-cholesterol ratio.

### 3.5. Stratified analysis

**Figure 5.**
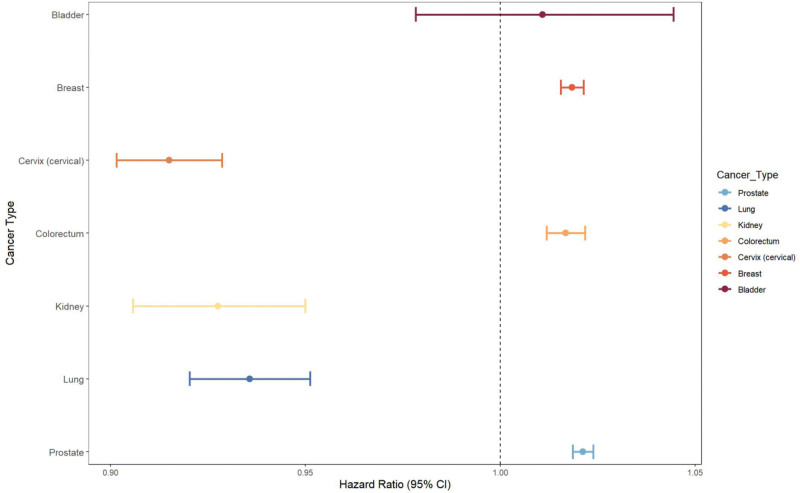
Forest plot for stratified analysis. CI = confidence interval.

By stratifying cancer types, we aimed to uncover differential associations of UHR and all-cause mortality in participants with different cancer types (those with multiple cancers, whichever was the 1st diagnosed cancer type) (Table 4, Fig. 5). Using weighted Cox proportional hazards regression models, a stratified analysis was carried out. To ensure statistical reliability, stratified analyses were conducted for the 7 most prevalent cancer types in our cohort (bladder cancer, breast cancer, cervical cancer, colorectal cancer, renal cancer, lung cancer, and prostate cancer), with all *P* values adjusted using the FDR method. The results revealed significant heterogeneity in the association between UHR and all-cause mortality among different cancer types. Specifically, in patients with breast cancers (HR = 1.02, 95% CI: 1.016–1.021), colorectal cancers (HR = 1.02, 95% CI: 1.012–1.022), and prostate (HR = 1.02, 95% CI: 1.019–1.024) cancers, the risk of death increased by approximately 2% for each 1-unit increase in UHR (*P*_adjusted < .001). In contrast, significant negative associations were observed in cervical, kidney, and lung cancers (HRs of 0.92, 0.93, and 0.94, respectively; *P*_adjusted < .001). No significant association was found in the bladder cancer subgroup (HR = 1.01, *P*_adjusted = .518).

**Table 4:** Stratified analysis of UHR and all-cause mortality among participants with different types of cancer.

Type	HR (95% CI)	*P*_raw value	*P*_adjusted value (FDR)
Bladder	1.01 (0.978 –1.044)	.5180	.5180
Breast	1.02 (1.016 –1.021)	<.001	<.001
Cervix (cervical)	0.92 (0.902 –0.929)	<.001	<.001
Colorectum	1.02 (1.012 –1.022)	<.001	<.001
Kidney	0.93 (0.906 –0.950)	<.001	<.001
Lung	0.94 (0.920 –0.951)	<.001	<.001
Prostate	1.02 (1.019 –1.024)	<.001	<.001

CI = confidence interval, FDR = false discovery rate, HR = hazard ration, UHR = serum uric acid to HDL-cholesterol ratio.

### 3.6. Sensitivity analysis

Based on previous analyses, we discovered that the correlation between UHR and all-cause mortality of cancer patients was more pronounced in older patients (≥ 65 years old). To further validate the robustness of this association and more precisely assess the role of UHR in the elderly cancer population, we excluded participants under 65 years of age (n = 932) and conducted the analysis solely in the elderly patient population. Consistent with the primary study, we used 4 multivariate Cox regression models (Table 5). The results showed that in Model 1, as UHR levels increased, the mortality risk for patients gradually increased (HR = 1.17, 95% CI: 1.06–1.30, *P* = .0038). Adjusting for age and gender (Model 2) and further adjusting for race and health insurance (Model 3), the association weakened slightly but was still statistically remarkable (Model 2: HR = 1.13, 95% CI: 1.01–1.26, *P* = .0321; Model 3: HR = 1.13, 95% CI: 1.01–1.26, *P* = .0292). Crucially, even after fully adjusting for cancer type in Model 4, the positive association persisted (HR = 1.13, 95% CI: 1.01–1.26, *P* = .0278). The mortality risk was substantially higher for patients in the top quartile of UHR (Q4) than for those in the lowest quartile (Q1), and this association remained robust across all models. A significant trend (*P* for trend < .001) was observed for the UHR quartiles in all models, further supporting the dose-response relationship between greater UHR levels and higher death risk.

**Table 5:** Sensitivity analysis of UHR with all-cause mortality by excluding patients under 65 years of age*.

All-cause mortality	Model 1			Model 2			Model 3			Model 4		
	HR	95% CI	*P*	HR	95% CI	*P*	HR	95% CI	*P*	HR	95% CI	*P*
UHR	1.17	1.06–1.30	.0038	1.13	1.01–1.26	.0321	1.13	1.01–1.26	.0292	1.13	1.01–1.26	.0278
Q1	Reference											
Q2	1.04	0.76–1.42	.8319	0.95	0.70–1.29	.7469	0.96	0.71–1.30	.8127	0.97	0.72–1.31	.8443
Q3	0.91	0.65–1.27	.5749	0.94	0.68–1.30	.6963	0.96	0.69–1.34	.8131	0.96	0.69–1.34	.8150
Q4	1.69	1.25–2.28	<.001	1.46	1.07–1.98	.0165	1.46	1.07–1.98	.0165	1.46	1.07–1.99	.0162
*P* for trend			<.001			<.001			<.001			<.001

Model 1 was left unaltered.

Model 2 had age and gender adjusted.

Model 3 had age, gender, race, and health insurance adjusted.

Model 4 had age, gender, race, health insurance, and cancer type adjusted.

CI = confidence interval, FDR = false discovery rate, HR = hazard ration, UHR = serum uric acid to HDL-cholesterol ratio.

* Four multivariate Cox proportional hazards regression models to control for confounding factors.

In order to further minimize the potential impact of cancer heterogeneity on the results, we excluded patients with multiple cancers (two or more types) (n = 259) and performed a 2nd sensitivity analysis. Patients with multiple cancers are usually accompanied by more complex metabolic disorders, chronic inflammatory states, and therapeutic interventions, which may lead to fluctuating UHR values and affect survival. It was observed that the association of UHR with all-cause mortality in single cancer individuals is notable. Among the unadjusted model, the HR was 1.24 (95% CI: 1.11–1.39; *P* < .001). After adjusting for age and gender, the HR decreased to 1.14 (95% CI: 1.02–1.28; *P* = .021). Further adjustment for race and health insurance, the HR remained unchanged (HR = 1.14; 95% CI: 1.02–1.28; *P* = .019). In the fully adjusted Model 4, which included cancer type, the HR was 1.14 (95% CI: 1.02–1.28; *P* = .0204), confirming the independent prognostic value of UHR. After categorizing into 4 groups (Q1–Q4) based on the UHR quartiles, the findings indicated that all-cause mortality tended to rise significantly with increasing UHR levels (*P* for trend < .05) and that patients in the Q4 group had a significantly higher risk of dying than those in the Q1 group. Consequently, UHR is further validated as an independent and robust predictor for all-cause mortality in cancer patients. See Table 6 for details.

**Table 6:** Sensitivity analysis of UHR with all-cause mortality by excluding patients diagnosed with multiple cancers (≥2)*.

All-cause mortality	Model 1			Model 2			Model 3			Model 4		
	HR	95% CI	*P*	HR	95% CI	*P*	HR	95% CI	*P*	HR	95% CI	*P*
UHR	1.24	1.11–1.39	<.001	1.14	1.02–1.28	.0213	1.14	1.02–1.28	.0190	1.14	1.02–1.28	.0204
Q1	Reference											
Q2	1.02	0.73–1.41	.932	0.84	0.62–1.15	.2814	0.84	0.62–1.14	.2689	0.85	0.62–1.15	.2941
Q3	1.12	0.80–1.57	.522	0.88	0.63–1.23	.4671	0.89	0.64–1.24	.4898	0.89	0.64–1.24	.4971
Q4	1.89	1.36–2.62	<.001	1.43	1.04–1.98	.0290	1.44	1.04–1.98	.0272	1.43	1.04–1.98	.0279
*P* for trend			<.001			.0046			.0039			.0045

Model 1 was left unaltered.

Model 2 had age and gender adjusted.

Model 3 had age, gender, race, and health insurance adjusted.

Model 4 had age, gender, race, health insurance, and cancer type adjusted.

CI = confidence interval, FDR = false discovery rate, HR = hazard ration, UHR = serum uric acid to HDL-cholesterol ratio.

* Four multivariate Cox proportional hazards regression models to control for confounding factors.

## 4. Discussion

For the 1st time, the association between cancer patients all-cause mortality and their UHR has been comprehensively evaluated. Using multivariate Cox regression models and Kaplan–Meier survival analysis, we discovered that increased UHR were significantly and nonlinearly related to the probability of mortality among cancer patients. Subgroup analyses further suggested that variables like age, gender, diabetes, and hypertension may modulate the association. Stratified analyses revealed the strong predictive power of UHR for all-cause mortality in breast, colorectal, and prostate cancer patients. These findings underscore that UHR may be a useful tool for assessing cancer prognosis and could be a significant predictor of cancer-related all-cause mortality.

UA is one of the purine metabolites,^[27,28]^ and has a complex relationship with cancer risk. On one hand, as a marker of oxidative stress,^[29]^ UA may play a role in the development of cancer by mediating DNA and cell membrane damage via oxygen radicals. On the other hand, its antioxidant effects may inhibit oxygen radical production,^[30]^ exerting potential anticancer effects.^[31]^ HDL-C is the main lipoprotein responsible for reverse cholesterol transport, which has antiinflammatory, antioxidant, and antiproliferative properties.^[32]^ It is discovered that low HDL-C has a positive correlation with elevated cancer risk.^[33]^ This may be due to the positive regulatory effect of HDL-C on inflammation-related diseases. Low HDL-C tends to impair the body antiinflammatory and immune regulatory capabilities, which in turn reduces cancer cell clearance. However, it has been shown that both abnormally high and low HDL-C levels may be linked to an elevated risk of cancer mortality.^[34]^ Therefore, neither single UA nor HDL-C level might be sufficient as an optimal predictor of cancer prognosis.

UHR is a novel biochemical marker introduced in recent years, which integrates information about inflammation and metabolic disorders, offering new perspectives for prognostic assessment in cancer patients. Elevated UHR levels have been demonstrated to be significantly linked to higher rates of morbidity and death in CVD and diabetes.^[35]^ Notably, these studies widely employed quartile-based stratification to identify high-risk phenotypes, confirming that this categorization methodology is scientifically accepted and clinically relevant for capturing the impact of metabolic dysregulation. It is widely known that CVD, diabetes, and cancer share similarities in terms of risk factors and pathologic mechanisms. For example, obesity and hyperlipidemia are 2 risk factors that are common to both cancer and CVD. Moreover, cancer and diabetes share similar mechanisms in metabolic regulation, such as insulin resistance and abnormal glucose metabolism.^[36]^ The above suggests that UHR might affect the onset and progression of multiple diseases through modulating shared biological pathways.

The prognostic validity of UHR in oncology is supported by strong biological plausibility, primarily reflecting the intersection of oxidative stress and lipid metabolism.^[37,38]^ In the tumor microenvironment, rapid cellular turnover generates excessive purines, culminating in hyperuricemia. Crucially, the intracellular accumulation of UA activates NADPH oxidase, exacerbating reactive oxygen species production and mitochondrial dysfunction.^[39]^ Furthermore, crystalline UA stimulates the NLR family pyrin domain containing 3 inflammasome, precipitating the release of pro-inflammatory cytokines (e.g., IL-1β and IL-18) that foster a chronic inflammatory milieu conducive to tumor proliferation and metastasis.^[40]^ In contrast, HDL-C counters these effects through systemic antiinflammatory and antioxidant activities, mediated largely by associated enzymes such as paraoxonase-1, which neutralizes lipid peroxides.^[41]^ In cancer patients, systemic inflammation frequently impairs HDL synthesis and suppresses paraoxonase-1 activity, critically attenuating these antioxidant defenses.^[42]^ Thus, elevated UHR signifies a synergistic state between these 2 pathways. This dynamic mechanism explains why the composite UHR outperforms individual biomarkers in predicting all-cause mortality.

As far as we are aware, this is the 1st study that systematically evaluates the relationship between UHR and all-cause mortality in individuals with cancer and explores its latent value as a cancer prognostic biomarker. The median follow-up period for the 2342 US individuals with 1 or more malignancies in this study was 81.0 months. These findings showed a strong correlation between raised UHR levels and a higher risk of all-cause death in cancer patients. Utilizing a quartile-based analysis, we observed that mortality risk is disproportionately concentrated within the highest UHR quartile (Q4), suggesting a critical threshold effect. RCS analysis delineated this inflection point at a UHR of 10.72. Clinically, this threshold serves as a potential benchmark. Specifically, maintaining a UHR below 10.72 correlates with a stable survival curve, whereas exceeding this value signals a transition into a high-risk metabolic state requiring intensive monitoring. Subgroup analyses revealed the predictive value of UHR levels for mortality, and this association was even more pronounced in patients over 65. This discovery is consistent with the evidence that UHR levels are strongly associated with morbidity and mortality from age-related diseases in persons over 50.^[40]^ Additionally, those with concomitant diabetes or hypertension showed a stronger correlation between higher UHR and mortality risk. This is likely due to the metabolic abnormalities in diabetic patients (such as hyperinsulinemia, high insulin-like growth factor I levels, hyperglycemia, dyslipidemia, etc), which may directly or indirectly promote cancer progression.^[38]^ Hypertension, on the other hand, has a prevalence of up to 37% of cancer patients and is recognized as one of the most common cardiovascular comorbidities, as well as one of the most frequent severe adverse events in cancer patients.^[43]^ Traditional cardiovascular disease risk factors (such as hypertension, diabetes, and obesity) may promote cancer cell proliferation and metastasis by altering the tumor microenvironment. These findings are consistent with the established comorbidity between metabolic diseases and cancer.^[44]^

Regarding the role of UHR in the prognosis of specific cancers, some studies have explored this topic. For example, hepatic steatosis score based on UHR and computed tomography values^[45]^ can effectively predict poor prognosis related to intrahepatic recurrence among individuals with colorectal cancer liver metastases.^[46]^ However, there are still insufficient studies exploring whether UHR could serve as a predictor for mortality risk across various cancer types or other specific cancers. The stratified analysis results revealed the heterogeneous role of UHR in different cancer types. Specifically, UHR was significantly and positively correlated with the risk of death from breast, colorectal, and prostate cancers, suggesting that UHR might be a positive indicator of mortality risk in patients with these cancer types. Notably, UHR was negatively correlated with mortality risk in individuals with cervical, kidney, and lung cancers, which may be explained by the following factors. First, tumor heterogeneity may be an important reason for this result. Tumors from different tissue origins have distinct biological characteristics and metabolic differences.^[47]^ For example, there is a substantial correlation between human papillomavirus infection and cervical cancer, and the pathogenesis of cervical cancer may be fundamentally different from other cancer types.^[48]^ Kidney cancer is particularly heterogeneous, with subtypes including chromophobe renal cell carcinoma, papillary renal cell carcinoma, and clear cell carcinoma, each of which varies widely in molecular features and clinical behavior.^[49]^ Secondly, certain exposure factors may be protective for specific cancers while acting as contributors to others. For instance, obesity is a contributing factor to breast cancer,^[50]^ whereas it may play a protective role in the OS of those diagnosed with lung cancer.^[51]^ Thirdly, potential confounders could have influenced the observed outcomes. The widespread application of cervical cancer screening might have altered the evolution of this disease.^[52]^ Kidney cancer often remains asymptomatic during its initial phase and is typically diagnosed at an advanced phase, which may affect the prognosis and treatment outcomes.^[53]^ Advances in lung cancer diagnostic techniques, such as early screening of pulmonary nodules, may reduce the mortality risk.^[54]^ These observations highlight the importance of considering cancer heterogeneity in studies. Despite significant heterogeneity observed across specific cancer subtypes, our overall multivariate analysis (Model 4) aimed to determine the systemic impact of UHR. By incorporating cancer type as an adjustment factor, we found that the overall association between elevated UHR and increased mortality risk remained significant across the broader cohort. This suggests that the metabolic dysregulation indicated by high UHR leads to poor prognosis through cross-tumor systemic mechanisms such as oxidative stress and inflammation, rather than being specific to any single cancer type.

To enhance the validity of the findings and solve the issue of multiple hypothesis testing, this study employed the FDR correction approach during the subgroup and stratified analyses. Compared with the traditional Bonferroni correction method, FDR can not only effectively control the false positive rate, but also reduce the risk of false negatives while maintaining high test efficacy. Particularly when multiple subgroups or variable groups are involved, FDR correction allows each hypothesis to be assessed for reasonable significance and thus avoids excessive correction,^[55]^ thereby enhancing the robustness of the analytical results and improving statistical credibility. Raw *P* values and FDR-adjusted *P* values are presented in all analyses. The adjusted *P* values showed some changes compared to the raw *P* values. However, in the BMI subgroup, the *P* values failed to maintain significance after adjusted (*P*_raw = .035, *P*_adjusted = .076), suggesting that support for the original hypothesis was weakened after controlling for multiple comparison errors. Moreover, the adjusted *P* values remained unchanged in the subgroup with PIR ≥ 3.76 and in the stratification of bladder cancer, possibly indicating that the significant associations between the variables were already strong enough on their own, with minimal change in the results after FDR correction. Even after multiple corrections, the relationship between UHR and mortality remained robust.

Although this study provides preliminary evidence for UHR as an indicator of all-cause mortality in individuals with cancer, certain limitations remain. First, as a cross-sectional study, we could not clarify the cause-and-effect relationship, and thus further longitudinal research is required to verify the relationship in the future. Second, although we adjusted for cancer site heterogeneity, the NHANES dataset lacks detailed cancer-specific clinical characteristics, particularly tumor staging, treatment modality, and time since diagnosis. These factors are key determinants of cancer prognosis^[56,57]^ and potential confounders. Future refined clinical data studies based on hospital cohorts are needed to validate the independent prognostic value of UHR after controlling for disease stage and treatment history. Third, the possibility of residual confounding cannot be entirely ruled out. Although we adjusted for comprehensive sociodemographic and clinical covariates, certain unmeasured lifestyle factors, such as detailed dietary intake, physical activity intensity, and alcohol consumption, were not fully captured due to data limitations. It is also worth noting that cancer survivors often undergo lifestyle modifications, such as strict alcohol restriction, following diagnosis; thus, baseline alcohol consumption recorded in the survey may not accurately reflect lifetime exposure. Similarly, specific inflammatory or metabolic diseases not explicitly screened for in NHANES may simultaneously influence both UHR and cancer prognosis. Future prospective studies utilizing more granular data are needed to minimize such confounding factors. Fourth, cancer diagnoses in the NHANES database rely on participants self-reports, which may result in misdiagnosis or missed diagnoses. More objective and reliable methods of cancer diagnosis, such as linkage to cancer registries or clinical pathology reports, should be considered in the future to strengthen the findings. Fifth, this study only analyzed baseline UHR measurements, while UHR levels may change dynamically during long-term follow-up. In the future, we must further assess the prognostic value of changes in UHR. Sixth, due to inherent sample size limitations in the NHANES survey design, our stratified analysis was restricted to the 7 most common cancer types. Consequently, this study cannot evaluate the prognostic value of UHR in malignancies beyond these 7. Future research utilizing large-scale, disease-specific cohorts is needed to validate the applicability of UHR across a broader spectrum of cancer types. Seventh, the mechanism of the link between UHR and mortality was superficially discussed in this study, future studies could explore animal models or cellular experiments to provide deeper insights. In addition, the lenient nature of the FDR correction method may result in some small effects not being significantly detected, while the risk of type II errors (false negatives) should not be ignored, especially in less significant variables (e.g., BMI). Future studies could further explore the applicability and comparative effectiveness of multiple correction methods to better balance false positive and false negative outcomes.

Although there are limitations, our study offers multiple advantages. First, we analyzed data from NHANES, a dataset with an extensive sample size, prolonged follow-up duration, and reliable data. Secondly, we employed a weighting methodology and conducted FDR correction, along with sensitivity analyses. The results aligned with the findings from primary study, enhancing the validity of the research. Finally, this study marks the 1st attempt to thoroughly assess the prognostic value of UHR among different cancer patient populations. Overall, this study emphasizes the potential significance of UHR as a prognostic factor for all-cause mortality in cancer patients, especially in the subgroups of elderly patients and those with comorbid metabolic diseases, which warrants further clinical attention. Further research ought to continue exploring the prognostic value of UHR across different cancer types, examine potential confounding factors, and validate these findings through large-scale prospective cohort studies. The ultimate goal is to assess the feasibility of UHR as an intervention target in cancer patients to improve survival outcomes.

## 5. Conclusion

This study reveals that high UHR levels are significantly correlated with all-cause mortality in individuals with cancer. Cancer patients are more at risk of dying if their UHR level is elevated. This finding highlights the latent clinical significance of UHR as a cancer prognostic predictor. Consequently, the development of targeted intervention strategies and the rational management of UA and HDL-C among cancer patients may be crucial in reducing adverse health outcomes.

## Acknowledgments

We thank the National Health and Nutrition Examination Survey participants and staff and the National Center for Health Statistics for their valuable contributions.

## Author contributions

**Conceptualization:** Jiaying Zhang, Dong Niu.

**Data curation:** Jiaying Zhang, Dong Niu, Valery Smirnov, Jiahui Li, Vladimir I. Gegechkori, Hengzhou Zhu, Xiaodan Zhu.

**Formal analysis:** Jiaying Zhang.

**Funding acquisition:** Chunhui Jin.

**Visualization:** Chunhui Jin.
